# Real world outcomes of Kahook Dual Blade goniotomy in black and Afro-Latinx adult patients with glaucoma: a 2-year retrospective study

**DOI:** 10.3389/fopht.2025.1573937

**Published:** 2025-06-16

**Authors:** Ayobami Adebayo, Chester Ng, Daniel Laroche

**Affiliations:** ^1^ Department of Ophthalmology, Albert Einstein College of Medicine, Bronx, NY, United States; ^2^ Advanced Eyecare of NY, Manhattan, NY, United States; ^3^ Department of Ophthalmology, Mount Sinai Eye and Ear, Manhattan, NY, United States

**Keywords:** Kahook, goniotomy, Afro -Latino, cataract, surgery

## Abstract

**Background:**

The Kahook Dual Blade goniotomy has been shown to be efficacious in the treatment of open angle glaucoma. We previously reported 6 months results using the Kahook Dual Blade in Black and Afro-Latino patients.

**Objectives:**

The purpose of this study was to determine the effectiveness and safety of Kahook Dual Blade (KDB) goniotomy alone or coupled with phacoemulsification cataract surgery to minimize intraocular pressure, number of medications used and visual field preservation in Black patients or Afro-Latinx who have open-angle glaucoma (OAG).

**Design:**

This was a retrospective, nonrandomized study that was carried out at two private practices in Harlem, NY and Queens, NY.

**Methods:**

This study consisted of patients with OAG who underwent phacoemulsification combined with goniotomy (PE + KDB) or goniotomy alone (KDB). The Kahook dual blade was used to perform goniotomy in all patients. Reduction of intraocular pressure (IOP) and alleviating the burden of medications were both considered indications for glaucoma surgery. Our research included information on IOP before and after surgery, the number of medications to decrease IOP pressure, visual field mean deviation, during a follow-up period of two years.

**Results:**

At two years we identified 31 patients who had surgery. The preoperative IOP of all 31 eyes which had surgery was 16.7 mmHg which decreased to 14.0 mmHg after two years. The baseline number of topical IOP-lowering medications was 2.4 ± 1.4 at baseline which decreased to 1.6 ± 1.4 (P = 0.02) after two years. The average visual field mean deviation was stable in both groups after two years. Postoperative adverse events were mild and included transient hyphema, IOP spikes, posterior capsule opacification, tearing, glare and mild pain.

**Conclusion:**

In Black or Afro-Latinx patients with open-angle glaucoma, phacoemulsification coupled with Kahook dual-blade goniotomy considerably reduces IOP and the number of medications.

## 1 Introduction

Glaucoma and cataract are the two most prevalent causes of blindness globally (1). The risk of developing glaucoma is significantly greater in the Black and Afro-Latinx groups of people (2). Cataract surgery and the reduction of IOP are important components of glaucoma IOP lowering therapy (3). The trabecular meshwork (TM), and Schlemm’s canal is responsible for the drainage of aqueous fluid from the eye, and as people become older, the trabecular meshwork thickens and Schlemm’s can decrease in size (4). There is a gradual increase in lens mass that occurs with age (5), as well as an anterior shift of the lens zonules next to the posterior iris. Both changes occur in conjunction with one another. The increased lens size will often cause Schlemm’s canal to become compressed, which in turn can lead to a rise in IOP (4). As the lens become larger, this brings the lens zonules closer to the posterior iris. Pigment obstruction of the trabecular meshwork is caused by the gradual release of pigment from the lens-iris contact that occurs with greater intensity due to the increased contact during accommodation and in individuals who have lengthy zonules (2, 5). This ultimately leads to an increase in the IOP of the eye.

In the Baltimore eye survey, the mean IOP in normal eyes was measured at 15 mmHg, whereas the mean IOP in glaucoma patients was measured at 18 mmHg (6). The most important aspect of therapy for glaucoma is lowering a patient’s IOP to protect the optic nerve and maintain normal visual function. The conventional treatment for this condition has been medical therapy, consisting of eye drops intended to reduce IOP. However, medical treatment for glaucoma is often ineffective for a large number of patients due to adherence (7). Treatment with eye drops can also contribute to worsening cataracts and increased lens thickness. Cataract surgery lowers IOP and medication burden in patients with glaucoma (8).

It has been shown that cataract surgery, can eliminate the iridolenticular contact and subsequent pigment liberation, and that can block the trabecular meshwork (9). Expansion of Schlemm’s canal also occurs with removal of the lens. In individuals with glaucoma, goniotomy removes the obstruction of the trabecular meshwork to restore outflow to Schlemm’s canal and bring down the IOP (10).

The Kahook Dual Blade (KDB) is a device that can be used to reduce IOP either on its own or in conjunction with cataract treatment (11). In comparison to the more conventional procedures of trabeculectomy and tube shunt implantation, KDB has a lower risk of complications that could be sight threatening (12).

There is a higher incidence of fibrosis in reaction to filtration surgeries, so there is an additional interest in evaluating their response to goniotomy, however, Black, and Afro-Latinx patients are often underrepresented in large clinical studies (2). The effectiveness of goniotomy with KDB was demonstrated in previous study by Laroche et al. as either an independent procedure or in combination with phacoemulsification. During the course of the research that lasted six months, the mean IOP was significantly reduced from 17.4 mmHg at baseline to 14.0 mmHg at month, a 19.5% reduction (11). This previous study reported six-month data in which the study started with 63 eyes. Here we report the follow-up data on two years in this group of patients. Many were lost due to lack of follow up due to a variety of reasons including lost or change of insurance and moving out of state. Eleven eyes also needed additional surgery.

In this article, we present the effectiveness and safety outcomes of goniotomy using the KDB with or without phacoemulsification for a period of two years in patients who had medically managed OAG alone and/or clinically significant cataracts to further determine its effectiveness in Black and Afro-Latino patients.

## 2 Methods

This is a retrospective report on patients with open-angle glaucoma who underwent KDB goniotomy alone if pseudophakic as well as combined cataract extraction, and KDB goniotomy (PE + KDB) if phakic. Every procedure that was carried out which included human participants were carried out in compliance with the ethical standards of the Icahn School of Medicine Institution Review Board. Given that this was retrospective research, the Institutional Review Board at the Icahn School of Medicine of Mount Sinai approved waiver for consent for the study. The data for this study came from a single practice in New York in the United States and all surgeries were completed by the same surgeon.

The Kahook Dual Blade (KDB; New World Medical, Rancho Cucamonga, California, USA) is a disposable ophthalmology knife manufactured by New World Medical that is intended for use in goniotomy procedures. The trabecular meshwork and the proximal section of Schlemm’s canal are removed as a result of this procedure. The justifications for goniotomy were to lower IOP and/or to decrease dependence on IOP-lowering medications at the time of cataract operation in the eyes of glaucoma patients.

The pre-and postoperative information was analyzed, and the clinical outcome was evaluated one day, one month, one year and two years after surgery. Postoperatively, the patient’s IOP objectives were individualized to each patient, and medications that reduce IOP were either stopped or added depending on what was thought to be essential.

The primary objective of this research was to determine whether there was a change in IOP from the preoperative baseline. The reduction in IOP, the quantity of IOP-lowering prescription required two years after the operation, and the visual field (Humphrey 24-2) were the three outcome variables that were investigated. The level of safety was determined by compiling a list of unfavorable events, both solicited and unsolicited, beginning with the intraoperative period and continuing through the final patient evaluation.

Descriptive statistics were used to summarize continuous variables as means with standard deviations, and categorical variables as counts and percentages. Paired t-tests were used to assess changes in intraocular pressure (IOP) and the number of medications from baseline to follow-up. A p-value of <0.05 was considered statistically significant. All statistical analyses were performed using Microsoft Excel and IBM SPSS Statistics.

## 3 Procedure

Goniotomy was carried out utilizing the KDB per the manufacturer’s instructions for its use. Goniotomy with resection of the TM and the inner wall of Schlemm’s canal was performed with the KDB. Combined with phacoemulsification, the 120-degree KDB goniotomy was conducted after the conclusion of cataract surgery with successful intraocular lens implantation. 120-degree Goniotomy with excision of the TM and the inner wall of Schlemm’s canal was performed with the KDB. To perform the KDB procedure, the temporal clear corneal incision that was left over from the cataract surgery was utilized. In cases where 120-degree goniotomy was conducted as an independent procedure, a temporal clear corneal incision was made with a keratome measuring 2.0 mm to create space for the KDB. The successive insertion of viscoelastic, treatment, irrigation and all came in through the same entry point. To enlarge the angle formed by the anterior nasal chamber, cohesive viscoelastic was injected into the anterior chamber. This decreases the anterior movement of the pupil, which can obstruct the view of the angle gonioscopically. After that, the head of the patient was turned approximately 45° in the opposite direction of the surgeon, and the microscope was angled approximately 45° in the surgeon’s direction.

To bring the TM into an en face view, a disposable direct gonioprism (Katena, Parsippany, New Jersey), was positioned on the corneal surface using the non-dominant hand. When inserting the KDB through the temporal clear corneal incision puncture and bringing the blade forward to the frontal angle, the right hand, which is the dominant hand, was used. The TM was engaged with the help of the tip of the KDB, while Schlemm’s canal was entered via the heel of the device. The blade was transferred from right to left within the canal so that the dual blades could produce parallel incisions along the TM. This resulted in the resection of a segment of tissue that measured about 120 degrees TM. Following that, the blade was extracted from the eye. After that, irrigation and aspiration were performed in the eye to eliminate residual viscoelastic.

All incisions were hydrated in order to attain a water tight closure. Pressurizing the eye at the end of the procedure to 15-20mmHG and instructing the patient to keep their head elevated for the first 4 days and nights by sleeping on 2–3 pillows helps to reduce hyphema.

Following surgery, patients were instructed to keep the head elevated above the waist for 4 days including sleeping on several pillows to accomplish this at night. The patients were also instructed to begin a course of treatment consisting of four doses of 1% prednisolone acetate suspension per day and four doses of 0.5% moxifloxacin solution per day for a period of one week. After one week, moxifloxacin treatment was terminated. All glaucoma medications were discontinued postoperatively and restarted only if needed to achieve the individuals target IOP. After continuing treatment for three weeks, prednisolone acetate was gradually reduced over the subsequent three weeks before being stopped altogether. In individuals who had cataract surgery and goniotomy conducted at the same time, the cataract surgery was done first using phacoemulsification. All the patients who had surgery were considered for inclusion. There was no upper age limit in place. There were no parameters for rejection based on a patient’s previous ocular history.

## 4 Results

This analysis looked at the remaining 31 different patients who had surgery (**Table 1**) from the initial 6 months report and had two-year follow-up. There were significant reductions in IOP with both KDB on its own and PE in combination with KDB (**Table 2**).

**Table 1:** Number of glaucoma medications at baseline and follow-up for all patients.

-	Pre - Operative	1 Year	2 Year
All Patients			
Eyes, n	31	31	31
No. Meds, mean (SD)	2.39 (1.3) *(95% CI: 1.91 to 2.87)*	*1.32 (1.3)* *1.32 (95% CI: 0.84 to 1.80)*	1.6129 (1.3) *(95% CI: 1.14 to 2.09)*
P value	N/A	0.001	0.02
KDB/phaco			
Eyes, n	27	27	27
No. Meds, mean (SD)	2.56 (1.3) *(95% CI: 2.05 to 3.07)*	1.33 (1.3) *(95% CI: 0.82 to 1.84)*	1.556 (1.4) *(95% CI: 1.00 to 2.11*
P value	N/A	0.0005	0.003
KDB Only			
Eyes, n	4	4	4
No. Meds, mean (SD)	1.25 (1.5) *(95% CI: -1.14 to 3.64)*	1.25 (1.5) *(95% CI: -1.14 to 3.64)*	2 (1.4) *(95% CI: -0.23 to 4.23)*
P value	N/A	1	0.6

**Table 2:** IOP at baseline and follow-up times for all patients.

-	Pre-Operative	Day 1	1 Year	2 Year
All Patients				
Eyes, n	31	31	31	31
IOP, mean (SD)	16.7 (6.13) *(95% CI: 14.45 to 18.95)*	10.6 (6.2) *(95% CI: 8.33 to 12.87)*	14 (6) *(95% CI: 11.80 to 16.20)*	*14 (5.7) (95% CI: 11.91 to 16.09)*
P value	N/A	0.0004	0.05	0.05
KDB/phaco				
Eyes, n	27	27	27	27
IOP, mean (SD)	17 (6.4) *(95% CI: 14.47 to 19.53)*	11.6 (6.0) *(95% CI: 9.23 to 13.97)*	14.7 (6.0) *(95% CI: 12.33 to 17.07)*	15 (5.6) *(95% CI: 12.78 to 17.22)*
P value	N/A	0.003	0.1	0.1
KDB Only				
Eyes, n	4	4	4	4
IOP, mean (SD)	16 (4.8) *(95% CI: 8.36 to 23.64)*	4.5 (4.8) *(95% CI: -3.14 to 12.14)*	10 (4.6) *(95% CI: 2.68 to 17.32)*	13 (7.3) *(95% CI: 1.38 to 24.62)*
P value	N/A	0.06	0.3	0.7

At the beginning of the study, the mean, standard deviation (SD) IOP for both eyes was 16.7 ± 6.1 mmHg. During the follow up after one year, the average IOP was 14.0 ± 6 mmHg (**Table 2**). During the follow up after two years, the average IOP was 14.0 ± 5.7 mmHg (**Table 2**). On postoperative day 1, a statistically significant decrease in IOP was detected. This decrease continued to be substantial at every postoperative time point during the first two years of follow-up. The IOP in every eye dropped by around 16.16%.

We found some differences in a second subgroup study in which patients who had both PE and KDB were compared to patients who had KDB alone. Only four eyes met inclusion criteria for the KDB-only group during the study period, and all were included in the analysis. At baseline, the patients who received the combined PE and KDB had an IOP that was 17 mmHg on average, with a standard deviation of 6.4 mmHg. These individuals had a reduction of 11.76% in their IOP from baseline at two years, when it averaged 15 ± 5.6 mmHg (**Table 2**). In this group, the mean amount of preoperative medication usage was 2.56 (standard deviation: 1.3) at the beginning of the study, but at the two years follow up mark, the number of medications had decreased to 1.556 (standard deviation: 1.4). This resulted in a considerable decrease of 1.004 medication(s) (39.21%) (**Table 1**).

At baseline, the patients who received KBD alone had an IOP that averaged 16 mmHg and had a standard deviation of 4.8 mmHg. At two years follow up, the average IOP was 13 ± 7.3 (representing a drop of 18.75% in IOP compared to baseline) (**Table 2**). During the two-year follow-up appointment, the mean number of IOP-lowering drugs was 2 ± 1.4, reflecting a 0.75 (60%) medication increase from the baseline, which was 1.25 ± 1.5 (**Table 1**). In this group that received KDB alone, there was no reduction in medication used.

Analysis of the patient’s visual fields showed that those in the PE + KDB group had a baseline average (SD) mean deviation of -7.66 ± 7.84 dB on Humphrey visual field (HVF), which remained steady during the two - year follow-up at -6.78 ± 8.15 dB. The KDB alone group had a higher HVF(SD) mean deviation of -21.46 ± 9.98 dB, which was stable at -21.3 ± 10.46 dB after two years (**Table 3**). The difference in baseline visual field MD in both groups is coincidental since at that time we offered KDB goniotomy to mild, moderate and advanced patients with glaucoma.

**Table 3:** Characteristics of patients undergoing Kahook Dual Blade goniotomy combined with phacoemulsification cataract surgery, and Kahook Dual Blade goniotomy alone.

-	Age, mean (SD)	% Male (n)	% Female (n)	CDR, mean (SD)	HVF – baseline (dB) mean (SD)	HVF – 2 Years (dB) mean (SD)
KDB/Phaco	69.4 (10.3)	44.7% (21)	55.3% (26)	0.7 (0.2)	−7.66 (7.84)	−6.78 (8.15)
Kahook	68.1 (14.7)	43.8% (7)	56.3% (9)	0.9 (0.1)	−21.46 (9.98)	−21.3 (10.46)

## 5 Discussion and conclusion

Similar to the previous study completed with six months follow up using the Kahook dual blade, this study was able to demonstrate that excisional goniotomy using PE + KDB and KDB alone was able to reduce IOP after two years. In addition, PE + KDB was able to reduce the number of medications used while preserving visual field at two years. Our previous study reported six-month data in which the study started with 63 eyes. However, some patients were lost due to lack of follow up due to a variety of reasons. Some of the cited reasons were change of insurance in addition to requiring additional and/or repeat surgery. 11 eyes out of 64 needed additional surgery so they were removed from the analysis. These 11 eyes that failed and required repeat surgery had an average pre-op VF MD of -15.02 ± 9.14. Thus in such advanced patients, consideration should be given to proceed to traditional glaucoma surgery.

Although our results demonstrated success in lowering IOP, our pressure lowering results were still slightly less than other studies which show up to 30% IOP reduction and a 50% reduction in medication. However, these studies did not include Black and Afro – Latino patients who have larger amounts of pigment present in the eye and suffer from increased scarring due to glaucoma surgery (13).

Many of the patients this study had moderate to advanced glaucoma on presentation (mean deviation on HVF: PE + KDB −7.66 ± 7.84, KDB alone −21.46 ± 9.98). We believe that the success seen in lowering IOP can be attributed to the removal of the juxta canicular trabecular meshwork restoring outflow to the collector channel; this effect is amplified when combined with cataract surgery.

A similar study performed by Mcniel et al. was also able to show favorable results using cataract surgery combined with goniotomy in an effort to reduce IOP and medication burden in patients (14). In their study they also compared different surgical cohorts, one cohort with cataract surgery alone and another with cataract/goniotomy surgery. After surgery, both cohorts showed a statistically significant decrease in IOP and medication burden when compared with preoperative baselines. In addition, cataract surgery combined with goniotomy was able to show a low incidence of IOP spikes. Out of 31 eyes, an IOP spike (IOP increase greater than 10 mmHg or IOP ≥ 30 mmHg) was seen in only one eye. Pressurizing the eye at the end of the procedure to 15-20mmHG and instructing the patient to keep their head elevated for the first 4 days and nights by sleeping on 2–3 pillows helps to reduced hyphema. Our study showed that received cataract surgery combined with cataract surgery, had a reduction of 11.76% in their IOP from baseline at two years in addition to a 39.21% reduction in the amount of ocular hypertensive medications used.

An important goal in the clinical management of glaucoma includes minimizing disability and visual field loss. Early goniotomy combined with cataract surgery can stabilize visual field and reduce medication burden. A study completed by Jackson et al. was able to show that about 1 in 8 eyes with glaucoma that receive routine care showed a fast progression in visual field loss at less than -1.0dB per year and that 1 in 3 eyes showed a visual field loss of less than -0.5dB per year (15). Their results suggest that new therapies are necessary to minimize functional disability in glaucoma patients that demonstrate fast progression. In our study, the PE + KDB group had a baseline average (SD) mean deviation of -7.66 ± 7.84 dB on Humphrey visual field (HVF), which showed an improvement after two years at -6.78 ± 8.15 dB.

It is safe to conclude that goniotomy combined with cataract surgery results in a decrease in IOP spikes, decrease in IOP and a decrease in medication burden. We do not recommend this in patient with advance glaucoma, but rather recommend traditional trabeculectomy or glaucoma tube shunt surgery. The inclusion of both combined Kahook with phacoemulsification and standalone Kahook goniotomy, as well as variability in the severity and subtype of OAG (e.g., NTG, JOAG, SOAG), may have introduced confounding factors that could influence the outcomes of this study. In the future, longer term follow up as well as randomized trials which include a larger number of Black and Afro Latino patients are needed to better assess the efficacy of minimally invasive glaucoma surgeries. In addition, more studies need to be performed to assess the combination of cataract and goniotomy surgery as a first line treatment for patients with glaucoma (2). Furthermore, while our study highlights the benefits of KDB goniotomy in Black and Afro-Latinx patients with OAG, it’s crucial to consider the role of genomic variations that may affect efficacy. For example, differential associations of genes like *MYOC*, *CDKN2B-AS1*, and *SIX6* with glaucoma risk and severity have been observed across different populations. Future research should investigate the prevalence and impact of such variations in glaucoma patients to potentially refine surgical outcomes and personalize treatment (16).

## Data Availability

The raw data supporting the conclusions of this article will be made available by the authors, without undue reservation.
